# Fibrosis in age-related neovascular macular degeneration in the anti-VEGF era

**DOI:** 10.1038/s41433-024-03308-6

**Published:** 2024-08-28

**Authors:** Beatriz G. Armendariz, Usha Chakravarthy

**Affiliations:** 1 Roche Pharma Research and Early Development, Roche Innovation Center Basel, 124 Grenzacherstrasse, 4058 Basel, Switzerland; 2 Honorary and Emerita Professor of Ophthalmology, Queens University of Belfast, Belfast, UK

**Keywords:** Macular degeneration, Eye manifestations

## Abstract

The natural history of neovascular age-related macular degeneration (nAMD) leads to scarring and loss of vision. Since the advent of anti-VEGF therapies, which are very effective for controlling exudation, large disciform scars are rarely encountered in the clinic. However long term studies show that smaller and less severe fibrotic scars are not uncommon and develop over time despite optimal treatment. This means that additional mechanisms of action may be required to completely address this condition. To permit new treatments, a proper understanding of the clinical impact of fibrosis is required. This review is focused on clinical aspects of fibrosis and summarises recent data on biomarkers, prevalence, causes, consequences, and therapies, highlighting the most important and urgent topics to tackle in order to advance in the treatment of fibrosis.

## Abstract (zh)

新生血管性年龄相关性黄斑变性 (nAMD) 的自然病程导致瘢痕形成和视力丧失。抗VEGF治疗, 因其控制渗出很有效, 在临床上已极少遇到大的盘状瘢痕。然而, 长期研究表明, 较小和不太严重的纤维化疤痕并不罕见, 即便采取了最佳治疗方案, 也会随着时间的推移而形成。因此可能需要其他机制来解释这一疾病。为推出新的治疗方法, 需要正确理解纤维化在临床中的潜在作用。本综述聚焦于纤维化的临床的生物标志物、患病率、病因、预后和治疗的最新数据, 强调为推进纤维化新的治疗而需要解决的最重要和最紧迫的问题。

## Neovascular AMD

**Fig. 1:**
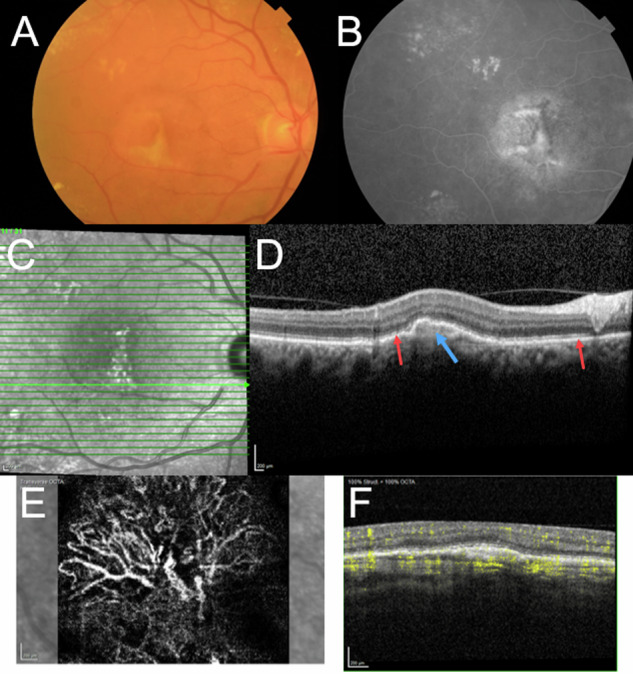
Multimodal images of the right eye of a patient with treated nAMD. **A** Colour fundus photograph showing an area of yellowish lesion of crescentic shape and partly defined edges surrounded by an area of pallor. **B** Late frame of a fluorescein angiogram showing staining of the area comprising the lesion with fading of hyperfluorescence in the area of pallor seen in **A**. **C** Volume scan of the macula with the B-scan shown in **D** highlighted in green. **D** The B-scan transects the inferior region of the lesion and external to the highly reflective RPE band (red arrows) a region of hyperreflectivity is visible (blue arrow). **E** En-face representation of flow in the neovascular membrane. **F** Corresponding B-scan, outer retina choriocapillaris slab showing flow signal in the segmented area.

Age-related macular degeneration (AMD) is a leading cause of vision impairment in older people. Its prevalence is expected to increase as the population ages and is predicted to affect around 288 million people in 2040 [1]. As its name indicates, AMD is a disease of aging that primarily affects the macula, within which lies the fovea which is responsible for resolution and hence for activities such as reading, driving and being able to see detail (Fig. 1).

Although the exact mechanisms leading to AMD are unknown, a combination of factors contribute to its pathogenesis- these include environmental insults and genetic predisposition that lead to a decreased capacity of the retinal pigment epithelium (RPE) and choriocapillaris to maintain homeostasis. Waste products accumulate in the subRPE (drusen) or subretinal (subretinal drusenoid deposits, SDD) spaces, and eventually RPE and photoreceptors (PR) and choriocapillaris are lost. Clinically, a region of atrophy sufficiently large (around 450 microns) and thus visible by fundus examination or by colour photography is referred to as geographic atrophy (GA). In a proportion of individuals, the ocular tissues attempt to repair the metabolic health of the retina through the growth of new vessels (neovascular AMD), which may represent a response to the hypoxic environment created by the defective oxygen supply from the choroid to the RPE and PRs [2]. Hypoxia stimulates the release of vascular endothelial growth factor (VEGF) and other cytokines by the RPE, and the subsequent growth of new blood vessels. Because these new vessels are leaky and friable, exudation of fluid and whole blood occurs leading to separation of the tissue layers and subsequently disorganisation and fibrosis. This exudative pathology is known as neovascular AMD (nAMD). It is unclear which specific factors lead to one or the other late-stage (i.e. GA or nAMD) outcomes, but both have a tremendous impact on people suffering from this disease [3].

### Section summary

AMD is a progressive disease linked to aging, influenced by genetic and environmental factors. It is characterized by the disruption of the homeostasis of the neurosensory retina, the RPE and the choroidal blood vessels that can result in cell death (GA). In a percentage of individuals, new blood vessels arise as an attempt to solve this situation (nAMD).

## Fibrosis is an end-stage wound healing response

Strictly speaking, fibrosis is a histopathological definition in which there is permanent deposition of extracellular matrix (ECM) components that include collagen, elastin, and others, as a result of tissue injury. Fibrosis is a stereotypical physiological response aimed at restoring anatomical integrity with minimal or no return of function. The functional impairment caused by permanent fibrotic tissue deposition is seen in a spectrum of diseases including interstitial lung disease, end-stage liver and kidney diseases, heart failure and nAMD [4]. Fibrosis is characterized by four stages: inflammatory, proliferative, matrix remodelling and wound quiescence [5]. In the proliferative phase, there is formation of provisional matrix that functions as a scaffold for permanent matrix deposition- this is often referred to as scar or fibrotic tissue.

Aging is a chronic process that leads to a reduced capacity of the human body to maintain homeostasis due to stromal and immune senescence [4], a process by which diploid cells arrest their cell cycle in an attempt to avoid genomic instability [6, 7]. As immune function is critical to tissue repair, aging-induced immunosenescence can lead to a greater fibrotic response compared to that seen in younger individuals [8].

In the context of an aging disease like nAMD, the different components that contribute to the early pathophysiology (e.g. neovascularization and leakage, disruption of the blood-retina- barrier, etc) are also instrumental in eventual fibrosis development [9]. This alteration of the homeostasis and the hostile microenvironment may induce epithelial-mesenchymal transition (EMT), a process by which epithelial cells de-differentiate into myofibroblasts and that constitutes a key step in the development of fibrosis [10, 11]. Blood constituents, an essential component of the provisional matrix, create the permissive environment for fibroblast proliferation and ultimately collagen deposition [5]. Thus, fibrosis could be considered an inevitable consequence of nAMD: ultimately, fibrosis is the attempt by the body to restore the outer blood retinal barrier.

In this review, we discuss the outcomes and conclusions from existing literature when the cited author has used the term fibrosis to describe findings of their own original research.

### Section summary

Fibrosis is a stereotypical response to tissue injury occurring in all tissues across the body, including the eye. Though inevitable, it can lead to unwanted consequences of loss of function.

## The anti-VEGF revolution

Prior to the anti-VEGF era, the clinical manifestation of the natural history of nAMD was the formation of a disciform scar and blindness [12–14]. Disciform degeneration was defined as “fibrous tissue proliferation beneath the pigment epithelium or retina” by Green in 1977 [15]. Fibrotic tissue was identified as one of the lesion components most likely responsible for vision loss [16].

In 2015, the first agent that could effectively treat nAMD was approved: ranibizumab (Lucentis, Genentech Inc., South San Francisco, CA, USA and Novartis AG, Basel Switzerland). Ranibizumab targets the vascular endothelial growth factor (VEGF), which plays a key role regulating angiogenesis and permeability [17]. The pivotal clinical trial MARINA [18] showed that ranibizumab not only prevented loss of vision in nAMD treatment- naïve patients (the natural history for these patients), but also, and for the first time ever in this disease, that their vision could recover, as shown by the 7.2 letters gained on average in the ranibizumab 0.5 mg dose group (the dose subsequently marketed) versus an average loss of 10.4 letters by the sham group. This represents a treatment effect of more than 17 letters (~3 ETDRS lines), which is highly significant. ANCHOR demonstrated an even more impressive treatment effect of 20.8 letters difference favouring ranibizumab vs verteporfin [19]. The results of both MARINA and ANCHOR together proved the benefit of ranibizumab in all types of choroidal neovascularization. However, the results outside clinical trials were modest, and studies conducted in real-world settings show that on average visual acuity reverts to pre-treatment levels [20, 21].

A clear benefit of the introduction of anti-VEGF treatment is the subsequent absence of disciform scars in the clinic. MARINA showed a reduction in the growth of fibrous tissue/ disciform scar with ranibizumab [22]- however a certain amount of fibrosis was still observed.

The main benefit of treating nAMD by inhibiting VEGF seems to be the ability to control the increased permeability of the neovessels that is responsible for the accumulation of fluid in the retina- this is manifested by the rapid and convincing reductions in subretinal fluid (SRF) and intraretinal fluid (IRF), detected as early as 7 days after the first injection [23]. The direct impact on blood vessels of the neovascular membranes of VEGF inhibition can be studied with OCT-Angiography (OCT-A). Inhibition of VEGF can lead to a change in the vascularization of the neovascular membrane [24–26]. Immediately after injection of an anti-VEGF there is apparent pruning of small vessels, leading to an initial reduction of the area of the MNV [27]. Therefore, blood flow within the system increases, and this leads to enlargement of the calibre of the main blood vessels (arterialization) [26]. Patients with mostly capillary MNV membranes seem to respond better to anti-VEGF treatment than those with mainly arteriolar MNVs [24].

The pruning effect of anti-VEGF on blood vessels leads to a slowing in the growth rate of the membrane compared to the natural history [22]. Despite this inhibitory effect the neovascular membranes can increase in size during treatment. For example, data from CATT showed increased lesion size at year 5, which was independently associated with worse VA [28]. The data is contradictory in real world data sets- a potential explanation being suboptimal treatment regimens and/ or the MNV subtype: MNV grows 1.24 mm^2^ per year in patients treated with a PRN scheme (IVAN) [29], but proactive treatment regimens such as the treat and extend posology has been shown to result in a decrease in lesion size.

Despite the obvious efficacy demonstrated overall in treated populations, the individual response to treatment is highly variable and still difficult to predict [30]. Some 50% of patients show persistence of disease activity (i.e. fluid, haemorrhage etc) [31]. This high inter-patient heterogeneity has been extensively demonstrated in clinical trials and in clinical practice and underscores the remaining unmet need in nAMD: the underlying disease is not completely tackled by inhibition of VEGF.

### Section summary

Prior to anti-VEGF, the natural history of nAMD led to disciform scars and blindness. The introduction of anti-VEGF dramatically reduced, but not completely abolished, the development of fibrosis. Anti-VEGF revolutionized the outcomes of nAMD leading for the first time to recovery of vision at a population level. However, there was a large interindividual variability in the response to these treatments.

## Why fibrosis matters: impact on vision

The human visual system is based on an exquisite interplay between the structure of the eye and the correct functioning of its cells. Thus, any alteration of these features may lead to vision impairment. The pathological changes of particular note are the replacement by fibrocytes and collagen of photoreceptor matrix, RPE and choriocapillaris. In addition, the contraction of the fibrous components leads to disorganisation and distortion of any residual neural and RPE elements. The consequence of such pathological processes is progressive visual impairment. In fact, AMD leads to blindness when left untreated, due to the development of atrophy and/or disciform scar. And even with intensive therapy, the main reasons for vision loss are atrophy and macular scar formation [16, 28, 32], underscoring the impact of fibrosis in functional outcomes.

The location of fibrosis with respect to the fovea has a clear impact on the vision outcomes. Depending on the distance of fibrosis to the fovea, it can be classified as subfoveal, juxtafoveal or extrafoveal. In a post-hoc analysis of HARBOR, the number of patients with extrafoveal only fibrosis who achieved gains of 15 or more ETDRS letters was larger versus patients with any subfoveal fibrosis [33]. This is unsurprising when considering that BCVA measures visual acuity at the foveal centre.

The impact of fibrosis on vision depends on its location within the retina: when there is deposition of material immediately external to the neurosensory retina and internal to the RPE, the interaction and communication between the photoreceptors and the RPE may be altered and can lead to the degeneration of the former with consequent visual decline. However, sometimes this fibrotic material can accumulate externally to the RPE (i.e. in the subRPE space). Interestingly, the effects on BCVA are not so severe in these cases as compared with subretinal fibrosis [34]. Romano et al found that eyes with sub-RPE fibrosis had better BCVA at baseline and even after 10 years of follow up compared to eyes with subretinal fibrosis, all of whom were managed with a PRN anti-VEGF dosing scheme [35], though similar results have been found following a treat-and-extend regime [34]. The cumulative incidence of subRPE fibrosis increased steadily, whereas subretinal fibrosis appeared more frequently during the initial 4 years of follow up and reached a plateau afterwards. A potential explanation could be that in cases where fibrosis is confined to the subRPE space, the integrity of the neurosensory retina is maintained. On the contrary, subretinal fibrosis implies a break in the blood retinal barrier leading to worse outcomes. And in fact, subRPE scars show better integrity of the PR layer (assessed by measuring the level of disruption of the external limiting membrane and the ellipsoid zone layers) compared to scars that have a subretinal component [34]. Additional evidence is found by the phenomenon of regression of type 2 MNV (i.e. subretinal) into type 1MNV (i.e. subRPE), which has been associated with better BCVA after anti-VEGF therapy [36]

Whether fibrosis impacts vision or not is determined by the status of the overlying PR and outer retinal layers. Fibrosis may be associated, thus, with atrophic processes. In fact, patients with fibrosis showed a higher percentage of RPE atrophy and Ellipsoid Zone (EZ) disruption [37], and reduced retinal sensitivity assessed with microperimetry (MP) [37–40]

### Section summary

Fibrosis in the outer retina arising secondary to neovascularisation in AMD has profound effects on central retinal function even though the exudative process may be controlled by anti VEGF treatments. Subretinal and subfoveal fibrosis have larger impact on vision loss than fibrosis located underneath the RPE or outside of the fovea.

## How is fibrosis diagnosed in the clinical setting?

Fibrosis is a histological term, but it is extensively used in clinical practice. Since the deposition of collagen and other histopathological features are not observable in real time, several imaging surrogates have been proposed. The traditional en face imaging modalities most commonly used are fluorescence angiography (FA) and colour fundus photography (CFP), with optical coherence tomography (OCT) introduced more recently. All have their advantages and limitations, which is the reason why a multimodal imaging approach is often preferred [41]. On FA, fibrosis is usually defined as a region of blocked early fluorescence and/or staining, with minimal or no leakage in the late angiographic frames. On CFP, the definition of fibrosis usually involves terms like well-demarcated mounds of yellowish white tissue. On OCT, the presence of a highly reflective material (HRM) located between the neurosensory retina and the RPE/ Bruch’s membrane is usually considered fibrosis, since this tomographic sign has shown to be correlated with fibrosis on CFP [42]. However, there is a lack of agreement on a unique definition of fibrosis and on what imaging modalities should be used (reviewed extensively in [41]). There are important discrepancies between the different imaging modalities: subretinal fibrosis may be over-diagnosed when using CFP and FA alone [43]. Fibrotic material located external to the RPE may not be visible on the gold standard of CFP that is used for reporting presence of fibrosis. A search in the current available literature highlights the inconsistencies in the definition of fibrosis arising as a consequence of differences in imaging modalities used as well as reporting of findings at varying times after treatment initiation. Thus, the present review does not suggest a new definition of fibrosis due to the lack of well-conducted, robust agreement studies

HRM located in the subretinal space (i.e. between the PR and the RPE) is called SHRM. SHRM is an OCT feature that has been proposed as a surrogate marker of the presence of fibrotic tissue on CFP. There is evidence that the presence of SHRM correlates with lower visual acuity [44–47]; however, studies usually included different types of SHRM that show differential rates of resolution in response to treatment. Prior to treatment initiation, SHRM is undefined, with fuzzy borders that show variable reflectivity. Undefined SHRM correlates well with the presence of fibrin on CFP [45]. After treatment, it either disappears (likely because it is composed mainly of resolvable elements such as proteinaceous fluid) or persists (due to unresolvable elements such as formed blood vessels, collagen fibres, etc). With resolution of the fluid components (sometimes referred to as SHE- subretinal hyperreflective exudation- [48]), the residual SHRM becomes more reflective with better defined borders (well-defined SHRM)In the treatment naïve state, distinguishing resolvable elements of SHRM from fibrosis is not possible with SD- OCT even though there may be some subtle differences in hyperreflectivity between these components. On OCT, the proportion of SHRM that is due to the fluid phase will resolve with treatment, but components SHRM can persist and thought to contain blood vessels, myofibroblasts and collagen representing both vascular and pauci vascular scars. Segmentation of all the B-scans in a volume scan when the areas of persistent SHRM are interspersed with regions of potentially resolvable material makes this task challenging. However future imaging developments including the use of polarization sensitive OCT may permit differentiation of such elements even in the treatment naïve state. The majority of SHRM that persists after the fluid phase of exudation has resolved correlates with yellowish areas of scarring on colour images, thus its presence on SD-OCT should not be considered a proxy for fibrosis at least until after completion of the loading phase[49].

In those eyes in which undefined SHRM evolved to a more defined HRM over time, this change correlated with the onset of scar and lower VA [42, 50]. But even well-defined SHRM has been shown to affect visual acuity in different ways, depending on the evolution of its inner boundary. Under treatment, approximately half of the eyes with SHRM at baseline can develop hyperreflective material boundary remodelling (HRM-BR), defined as the appearance of a well-defined hyperreflective inner boundary that separated persistent HRM from the neurosensory retina continuous with the adjacent RPE layer. The development of this HRM-BR is associated with visual acuity gain similar to the gains obtained when SHRM completely disappears [51]. The nature of this hyperreflective band is unknown, as no histopathological correlates have been performed, but it could represent RPE cells.

Similarly to the evolution of fibrosis under VEGF treatment (section Why fibrosis matters: impact on vision), HRM external to the RPE is infrequent at baseline but increases to approximately one third by month 12 of anti-VEGF treatment [45]. HRM in the subRPE space seems to have less of an impact on vision than when HRM is subretinal [41, 45]. When HRM is sub RPE, photoreceptors may survive but long studies suggest that over time atrophy develops [52].

A promising new imaging modality that has the potential to increase our understanding of fibrosis dynamics is polarization-sensitive OCT (PS-OCT). The signal obtained with PS-OCT is based on the refringence properties of the imaged tissue. The presence of highly organized collagen fibres, a hallmark of fibrosis, results in a specific refringent pattern. This can be useful for the differential diagnosis of SHRM composition: non-fibrotic components of SHRM (such as blood, MNV membranes or fibrin) will not induce this birefringent signal on PS-OCT. As such, PS-OCT has been proposed as a more accurate technique to differentiate SHRM with respect to the RPE and other hyperreflective features [38, 53, 54].

### Section summary

The definition for fibrosis was derived during the era when clinical imaging merely consisted of colour fundus photographs and fluorescein angiograms. With the advent of high-resolution SD-OCT additional features that correlate with the characteristics of fibrosis on the previously available imaging modalities have been described. However, SD-OCT features that are now visible and described in chronic nAMD may not only reflect fibrosis but also other morphological changes such as thickened and distorted tissue layers and vascular structures. Hyperreflective material that lies external to the RPE while visualised by SD OCT is often not seen on colour or FA and its presence while inimical to visual function is not as profound as HRM located in the subretinal space. Therefore, a more accurate characterization of the extent and location of HRM on SD OCT and its correlates on the established imaging modalities of colour and FA is needed for a better and more accurate definition of fibrosis in the outer retina.

## Fibrosis by numbers

How frequently fibrosis develops is one of the most important questions that remain unanswered, likely due to differences in definitions and imaging modalities used, but also in patient population and treatment regimens of the different studies [41]. In a recent meta-analysis by Cheong et al, the prevalence of fibrosis at baseline, 12, 24, and 60 months was 13%, 32%, 36%, and 56%, respectively, considering real-world and clinical studies [55]. The overall incidence rate was 14.71 events per 1000 eye-months, meaning that fibrosis will develop in approximately 15 out of 100 eyes followed for 10 months. The studies that contributed to the reporting of the rates of fibrosis, however, showed a large heterogeneity.

Two studies have shown that fibrosis progressively develops with time. In a retrospective study that followed 207 patients during 10 years, only 2.2% of eyes show fibrosis at baseline, but the cumulative incidence at 10 years was of 62.7%, with an incidence rate of 8.9 per 100 person-years [35]. The Fight Retinal Blindness [56] registry also has 10 years follow up data, however they report lower prevalence of fibrosis (40.7% at 10 y). Of note, fibrosis was graded by physicians and only from April 2016, several years after the registry started.

### Section summary

The large variability in the incidence and prevalence of fibrosis, likely due to the lack of an agreed definition, impedes the establishment of the real unmet need of this disease.

## Classifications of fibrosis

There is no accepted severity classification of fibrosis- instead, several definitions based on different imaging modalities have been suggested [41]. Some of them focus on the extent of the lesion [16, 42, 57–59], and others on its severity [60–62].

A comprehensive analysis of OCT features in fibrosis based on location with respect to RPE and RPE integrity was published in 2020 by Souied et al. [63]. The lesions could be either subRPE (type A), mixed subretinal/ subRPE (type B) or not classifiable due to disruption of RPE layer (type C). They also proposed potential pathways of fibrosis progression. Their suggestion, although comprehensive, was retrospective and based on a limited sample size, and has not been widely used by the community.

A classification of fibrosis progression, stages and severity will be useful for the design of clinical trials focused on finding a treatment for fibrosis, as studies such as these will be needed in order to show efficacy of the intervention. However, before a sensitive and validated classification of the severity of fibrosis is developed, agreement on the definition of fibrosis is required. At present there is no consistency of language in the definition of fibrosis even within individual imaging modalities.

A classification of fibrosis should include descriptive imaging biomarkers that are correlated with onset, severity and extent of fibrosis, and should take into account its dynamic nature, establishing the ideal time point for fibrosis evaluation.

### Section summary

Similarly to the lack of agreement of a fibrosis definition, there is no classification of fibrosis that could allow the evaluation of any potential therapeutic.

## Fibrosis in the anti-VEGF era

Although, as discussed in section Fibrosis is an end-stage wound healing response, large disciform scars are rare after the introduction of anti-VEGF therapy, fibrosis is detected in patients with nAMD. To which extent, though, has been difficult to determine, as there are important differences in definition and imaging modalities used (see section Fibrosis by numbers). In this section we explore the main reasons that could explain why fibrosis still develops despite adequate treatment.

### Risk factors: association vs causation

Several studies have identified biomarkers that predict the formation of fibrosis under anti-VEGF treatment. We have summarized their key observations in Table 1 and below:

**Table 1:** Examples of major risk factors identified for fibrosis development in patients with nAMD following intravitreal injections.

Increased risk of fibrosis	Comparison	References
**MNV type**		
Classic/predominantly classic MNV	Minimally classic and/or occult MNV	[32, 33, 56, 61, 67]
**MNV size**		
MNV area ≥4 DA	MNV area ≤1 DA	[67]
MNV area ≥5 DA	MNV area <1.5 DA	[61]
Larger lesion size per 1000 um	–	[56]
**Baseline demographic**		
Baseline BCVA ≤ 50 letters	Baseline BCVA ≥ 50 letters	[35]
Baseline BCVA ≤ 40 letters	Baseline BCVA ≥ 70 letters	[61]
Baseline BCVA ≤ 35 letters	Baseline BCVA ≥ 70 letters	[56]
Baseline VA < 20/100–160	Baseline BCVA ≥ 20/25-40	[67]
Fellow eye VA ≥ 20/20	Fellow eye VA < 20/50	[32]
Diagnosis-treatment interval ≥15 days	Diagnosis-treatment interval <15 days	[61]
**Treatment response**		
High-active MNV lesion	Low-active MNV lesion	[56]
Higher cumulative number of injections	–	[35]
**CF findings**		
Haemorrhage >1 DA	No haemorrhage	[32]
Submacular haemorrhage at any time point	No haemorrhage	[35]
**OCT findings**		
CST variation	No variation	[35]
SD quartile 4 FCPT	SD quartile 2 FCPT	[75]
foveal retinal thickness >212 μm	Retinal thickness <120 μm	[67]
foveal SRF or foveal SRF thickness >25 μm	No SRF	[32, 67]
No SRF	SRF	[68, 69]
IRF	No IRF	[66]
PED	No PED	[66]
No PED	PED	[56]
No RPE elevation	RPE elevation	[32, 67]
Foveal subretinal tissue complex thickness >275 μm	Thickness <75 μm	[67]
SHRM	No SHRM	[67]
No RPE atrophy	Localized RPE atrophy without lesion	[66]
No photoreceptor loss	Photoreceptor loss	[66]
Refractory IR cysts	Refractory SRF	[90]
Subretinal fibrovascular tissue	Absent	[91]

For a recent and comprehensive review please check Cheong et al. [55].

*BCVA* best corrected visual acuity, *CF* colour fundus, *DA* disc area, *FCPT* foveal centre point thickness, *IRF* intraretinal fluid, *MNV* macular neovascularization, *OCT* optical coherence tomography, *PED* pigment epithelium detachment, *RPE* retinal pigment epithelium, *SD* standard deviation, *SHRM* subretinal hyperreflective material, *SRF* subretinal fluid, *VA* visual acuity.

An important risk factor for the development of fibrosis is the presence of a subretinal lesion component (i.e. type 2 or a mixed MNV with some type 2 component). Per definition, a type 2 MNV arising from the choroid has disrupted the RPE to ramify within the subretinal space [64]. The components of this type of lesion are thus in direct contact with the apical microvilli of the RPE- potentially involved in EMT and fibrosis [10, 64, 65]. In contrast, a type 1 MNV has not yet led to a disruption of the RPE; these types of lesions have in fact been associated with less risk of fibrosis [33]. It is important to note, however, that fibrosis is not exclusive of type 2 MNV, and it can appear in all MNV types [33, 63]

Fibrosis is also associated with the presence of IRF [66]. The association of SRF with clinically obvious fibrosis is less clear. The presence of SRF has been associated with scar formation in some studies [67], whereas in others, including some real-world studies, it has been shown to lead to lower prevalence and reduced progression of fibrosis [68, 69].

PEDs are characterized by separation between the RPE and the inner most aspect of Bruch’s membrane. The space created by this separation is occupied by blood, serous exudate, drusenoid material, fibrovascular tissue or a combination of the above. The presence of a PED does not necessarily equate to fibrotic material as serous fluid and whole blood can also accumulate in this compartment in chronic nAMD. Thus, it is important to distinguish between the types of PED (i.e. serous, drusenoid or fibrovascular), as they have a differential impact on both persistence and or development of fibrosis [70, 71].

The role of PEDs in general as a risk factor is unclear: while some indicate elevated risk [66], others have shown that it is associated with reduced risk of fibrosis. For example, the FRB! 10- year cohort with PED at baseline had lower risk of developing fibrosis than those patients without PED [56]. It is hypothesized that in PED, the blood-retinal barrier is not impaired, and fluid therefore remains trapped beneath the RPE, in a similar way to type 1 MNVs.

Fibrovascular PEDs (fvPED) often have been likened to subRPE fibrosis. It can be hypothesized that they represent different stages of the spectrum of a Type 1 MNV indicating chronicity: initially, the neovessels represent the major component of the membrane. As the vascular components mature, the presence of fibrotic components increases. Subsequently mature, stable vessels with no leakage and with a predominance of fibrotic material become identifiable as multilayered PED [72]. Multilayered PEDs are composed of a subretinal pigment epithelium inhomogeneous hyporeflective space (layer 1), a hyper-reflective band beneath layer 1 (layer 2), and a hyporeflective space or prechoroidal cleft between the Bruch’s membrane and layer 2 (layer 3) [73]. The hyperreflective band. Fibrovascular PEDs show poor response to anti-VEGF treatment [70]. However, the visual acuity of patients with subRPE fibrosis is usually better than eyes with SHRM [35, 72, 74]. These seemingly contradictory results may be explained by several aspects: for example, the status of the outer neurosensory retina will greatly determine visual outcomes [72], however it is not systematically considered in most of the studies. In addition, very rarely are the results reported by PED type. Importantly, the lack of consensus on a fibrosis definition may be behind some of the discrepancies of the impact of SRF and PED on fibrosis.

Most studies merely report the impact of presence/ absence of specific biomarkers. Recent data uncovered the impact of an interesting novel aspect: notably, the dynamics of the resolution of fluid in the different retinal tissue compartments appear to play a role in fibrosis development. Variations in the thickness or volume of the retinal layers are detected even in studies with fixed regimens like CATT and IVAN. Patients who show larger fluctuations in quartile 4 of the standard deviation of the foveal centre point thickness had increased odds of fibrosis development (1.95 (95% CI, 1.42–2.68)) compared to eyes in quartile 2 [75]. High SRF variations as well as larger SHRM volumes were associated with worse BCVA results [76]. These outcomes could be explained by the attenuation of the PR layer [76] - which reinforces the importance of assessing the status of the neurosensory retina.

In agreement with this, patients with frequent disease reactivations, manifested as large CST variations, show fibrosis more commonly [35], and highly active lesions is associated with the presence of subretinal fibrosis [56]

### Causes of fibrosis during treatment

The development of fibrosis has been associated with less-than-optimal treatment [77]. The association between treatment frequency and fibrosis development was shown in the FRB registry: the Swiss cohort received half the injections of the Australian/ New Zealand (ANZ) cohort during follow-up (years 3–7). These resulted in worse visual outcomes in the Swiss cohort [78], including higher rates of fibrosis. These results suggest that a reduced frequency of injections leading to higher risk of fibrosis could be behind the worse outcomes observed in real world settings with anti-VEGF therapy as compared with clinical trials. Maintaining the suppression of free VEGF levels requires frequent injections [79]. Thus, fewer injections can lead to larger variations in the concentration of ocular VEGF, which could result in bursts of disease activity. The recurring presence of fluid and blood has deleterious effects on the homeostasis of the retina. If this hypothesis is true, then we could expect that longer acting devices such as the recently approved Susvimo (ranibizumab injection, Genentech Inc., South San Francisco, CA, USA and Novartis AG, Basel Switzerland) platform may result in a lower frequency of fibrosis.

A post-hoc analysis of MARINA [22] focused on the angiographic features of the neovascular membrane showed that scar tissue did not grow as much in the anti-VEGF arms vs sham arm; however, some fibrosis was still present. This was further corroborated by several randomized control studies (CATT, IVAN, HARBOR): Scar tissue was detected in approximately half of the patients after two years of intensive treatment [32] Fibrosis, together with atrophy, were identified as the components of neovascular membrane more strongly correlated with lower vision [16]. These results suggest that the inhibition of VEGF alone is not enough to prevent fibrosis development.

### Section summary

Risk factors for fibrosis include the presence of IRF, SRF and PED, though the results are sometimes contradictory. Not only their presence/ absence, but also dynamics of resolution may have an impact on the risk for fibrosis. Further research is needed.

## Clinical research focused on fibrosis

The fact that anti-VEGFs have significantly reduced both the severity and frequency of fibrosis development in patients with nAMD proves that at least some aspects of this pathology can be addressed by inhibiting VEGF. However, fibrosis develops even in nAMD eyes treated frequently with anti-VEGF, so other mechanisms of action are needed to fully address this condition. Understanding the molecular pathophysiology of fibrosis is key to develop targeted therapies [80, 81].

Currently there is no specific preventive treatment for fibrosis in nAMD. However, there have been several attempts to tackle it, albeit with disappointing results.

Pegpleranib (Fovista,Ophthotech, NY, US), a pegylated aptamer targeting platelet-derived growth factor (PDGF)- B, was the first asset positioned as an anti-fibrotic treatment for nAMD. PDGF is an essential molecule for the survival, recruitment and maturation of pericytes, which cover endothelial cells of the blood vessels. The hypothesis was that by eliminating this pericyte cover, the blood vessels would be more accessible to anti-VEGF treatment and thus lead to better treatment outcomes. Preclinical evidence in mice had showed that pegpleranib effectively inhibited fibroblast proliferation, epiretinal formation and retinal detachment [82]. The molecule, in combination with anti-VEGF, then entered clinical development. The phase 1 study [83] showed no safety findings, and thus proceeded into phase 2a (NCT02214628) and 2b (NCT01089517) trials. In these studies, the primary endpoint of BCVA was met [62]. The evidence of a potential anti-fibrotic effect came from a retrospective analysis using CFP, that showed that eyes treated with the highest dose of pegpleranib + anti-VEGF had less incidence, progression, and severity of fibrosis at week 24 compared to anti-VEGF alone. However, none of these results could be replicated in the extensive phase 3 program conducted (NCT01944839, NCT01940900, NCT01940887). The primary endpoint of mean change from baseline in BCVA was not met, thus leading to the discontinuation of the development of pegpleranib [84]. Since the results of the phase 3 studies have not been peer-reviewed published, it is not possible to definitively conclude the reasons for this lack of efficacy, however we now know that pericytes are necessary for a healthy microcirculation [85], raising questions about the impact of depleting them.

Ribomic (RIBOMIC Inc, Tokyo, Japan) is developing umedaptanib pegol (RBM-007), an RNA aptamer against fibroblast growth factor 2 (FGF-2). It is hypothesized that FGF-2 may be involved in neovascularization and the formation of fibrotic scar, and preclinical results show that the RBM-007 aptamer can lead to reduced CNV area and severity of submacular fibrosis in laser-induced CNV models [86]. The Phase 1/ 2a SUSHI study (NCT03633084) showed evidence of reduction in retinal fluid and SHRM reabsorption with an acceptable safety profile [87]. The molecule thus advanced to the phase 2b program with TOFU (NCT04200248) and its open label extension RAMEN (NCT04640272) studies, assessing umedaptanib pegol monotherapy and in combination with aflibercept (EYLEA, Regeneron Pharmaceuticals, Inc, NY, US), compared to aflibercept monotherapy, in previously treated nAMD patients. An additional study, the investigator-initiated Phase 2 TEMPURA study, focused on naïve patients (NCT04895293). Initial TOFU results have shown that umedaptanib pegol did not improve vision compared to aflibercept monotherapy, but all treatment arms showed cessation of disease progression, with pre-existing fibrosis remaining stable without worsening [88]. Preliminary data from TEMPURA showed that treatment with umedaptanib pegol is more effective in treatment naïve eyes. The company is planning to conduct further studies in naïve patients as well as in coordination or combination with anti-VEGF drugs.

Isarna (Isarna Therapeutics GmbH, Munich, Germany) is investigating the role of transforming growth factor beta (TGF-b) in retinal disease. This molecule has been extensively implicated in fibrotic processes in several organs, and there is some evidence suggesting that certain gene variants in the TGF-b pathway lead to more severe forms of AMD. The lead candidate, ISTH0036, is a locked nucleic acid-modified antisense oligonucleotide selectively targeting the messenger RNA (mRNA) of TGF-β2. Preclinical studies showed decreased neovascularisation and fibrosis development in murine models of CNV [89]. In the phase 1 study, a single intravitreal injection of ISTH0036 was administered to patients after trabeculectomy, with no major safety signals [89]. Following these results, the first patient was enrolled in the Phase 2a BETTER study in 2021, The BETTER study is an ongoing parallel, two-segment phase 2 open label clinical study to evaluate ISTH0036 in patients with nAMD and diabetic macular oedema (DME). The study aims to enrol 30 patients for each indication with a primary objective of reduction of retinal fluid and central macular thickness after 7 months of treatment. A key exploratory outcome will be the prevention of fibrosis and EMT.

### Section summary

Despite the potentially devastating effects in vision of fibrosis in nAMD, there is no treatment approved yet. Several companies have tried to address this, without any success up to now. A thorough understanding of the molecular pathophysiology and the role of the different cells involved is required for the development of an effective and safe treatment.

## Inferences & what’s next?

1. Development of fibrosis during treatment has not been solved. To date there are no treatments that specifically target fibrosis in the outer retina
2. Subretinal fibrosis is associated with worse function. There is a wealth of evidence indicating that presence of fibrosis defined using traditional colour and or FA imaging is associated with a very significant impact on visual acuity.
3. Persistent HRM may be considered a proxy for fibrosis, but robust agreement studies are lacking. Emerging data suggest that persistent HRM when localised to the sub RPE region only is not easily imaged using established imaging modalities of colour and FA and therefore the impact of sub RPE fibrosis has been underestimated.
4. When HRM is subretinal the outer retinal layers are often missing in that area and the photoreceptors are destroyed. When HRM is sub RPE, photoreceptors may survive but long studies suggest that over time atrophy will develop even when exudation due to new vessel invasion has been controlled in optimal fashion. These observations suggest that fibrosis even when located external to the RPE can lead to atrophy of the overlying PR because the fibrotic material likely impedes transfer of nutrients to PR from the choriocapillaris.
5. Current strategies to mitigate fibrosis include preventing episodes of acute recurrence of exudation. However, to date there are no therapies that might restore sick RPE function, nor do we have treatments that might prevent laying down of collagen, remove existing collagen, and/ or offer neuroprotection.
6. An agreed definition of fibrosis that includes imaging modalities, detection thresholds, progression and biomarkers is urgent. Without it, it is impossible to understand the true extent of the condition or to develop therapies to address it.
